# NOS2 Polymorphism in Aspect of Left and Right-Sided Colorectal Cancer

**DOI:** 10.3390/jcm13040937

**Published:** 2024-02-06

**Authors:** Justyna Klusek, Piotr Lewitowicz, Ruslan Oblap, Ewa Orlewska, Bartosz Witczak, Michał Tomasz Marzec, Monika Kozłowska-Geller, Łukasz Nawacki, Monika Wawszczak-Kasza, Kamila Kocańda, Artur Jóźwik, Stanisław Głuszek

**Affiliations:** 1 Collegium Medicum, Jan Kochanowski University, 25-317 Kielce, Poland; lewitowicz@gmail.com (P.L.); roblap@hotmail.com (R.O.); eorl@ujk.edu.pl (E.O.); witczak01@gmail.com (B.W.); monika.kozlowska.chir@onet.pl (M.K.-G.); lukasz.nawacki@ujk.edu.pl (Ł.N.); wawszczak.monika@gmail.com (M.W.-K.); kamila.kocanda@ujk.edu.pl (K.K.); sgluszek@wp.pl (S.G.); 2 Holy Cross Mother and Newborn Provincional Centre, 25-371 Kielce, Poland; 3 Department of Biomedical Sciences, University of Copenhagen, 2200 Copenhagen, Denmark; mimarzec73@gmail.com; 4 Institute of Genetics and Animal Biotechnology, Polish Academy of Sciences, Jastrzębiec, 05-552 Magdalenka, Poland; aa.jozwik@igbzpan.pl

**Keywords:** colorectal cancer, NOS2, gene polymorphism right-sided CRC, left-sided CRC

## Abstract

**Background**: The NOS2 gene polymorphism rs2297518 is associated with an increased level of NO, which could contribute to colorectal cancer (CRC) development. We hypothesized that the potential influence of the NOS2 gene polymorphism on cancer development may vary between right-sided and left-sided colon cancers, and rectal cancers. The aim of this study was to determine the rs2297518 polymorphism influence on colorectal cancer development with regard to tumor localization. **Methods**: This case–control study included 199 patients with CRC and 120 controls. The qPCR endpoint genotyping was conducted using the TaqMan^®^ genotyping assay. **Results:** This study revealed significant differences in tumor characteristic and in the minor alelle A frequency in the NOS2 genotype between colorectal cancers with different localizations. The mucinous adenocarcinoma was diagnosed significantly more often in right-sided cancers than in left-sided (30.6% vs. 10.9%, *p* = 0.009) and rectal cancers (30.6% vs. 7.1%, *p* = 0.0003). The minor allele A of the NOS2 genotype was observed more frequently in right-sided cancers than in left-sided cancers (44.9% vs. 23.1%, *p* = 0.0137) and more frequently in rectal cancers than in left-sided cancers (40.0% vs. 23.1%, *p* = 0.0285). **Conclusions:** In conclusion, the results support the hypothesis that the SNP rs2297518 of the NOS2 gene influences colorectal cancer development with regard to tumor localization.

## 1. Introduction

Colorectal cancer (CRC) is a multifactorial disease with proven diet, local inflammation and environmental-related genesis. The molecular pathway frequently varies between left- and right-sided tumors and is also tightly connected to tumor morphology. The single-gene mutation burden significantly influences cancer development, particularly in the hereditary CRC predisposition syndromes, such as familial adenomatous polyposis (FAP) and hereditary non-polyposis colorectal cancer (HNPCC). Genes with low penetrance have the potential to notably modify the susceptibility to carcinogenesis, by altering the body’s vulnerability to carcinogens or affecting an individual’s oxidative stress levels [1]. The oxidative balance of the body is determined by both diet and lifestyle, as well as genes associated with pro- and antioxidative processes within cells. The imbalance between pro- and antioxidants leads to oxidative stress, manifested by elevated levels of reactive oxygen species (ROS) and reactive nitrogen species (RNS). These, through DNA damage, contribute to heightened genomic instability. That is, the mechanism by which cancer initiation and progression have been linked to oxidative stress [2].

One of the RNS is NO (nitric oxide), a short-lived, small molecule generated by nitric oxide synthase (NOS) [3]. There are three types of NOSs in mammalian systems. Two of them are constitutively expressed in neurons (nNOS coded by the NOS1 gene) and epithelial cells (eNOS coded by the NOS3 gene) but produce relatively low amounts of NO (nM levels) in a process that is regulated by Ca^2+^ binding to calmodulin [4,5]. The third one, inducible NOS (iNOS) coded by the NOS2 gene, is expressed in inflamed tissue in response to pro-inflammatory cytokines [6,7]. It can produce larger amounts of NO than other NO synthases together (µM levels) in a calcium-independent manner. iNOS is one of the most important enzymes involved in the metabolic pathway of reactive oxygen and nitrogen species during inflammation. NO plays an important role in vasodilation, smooth muscle relaxation and immunity [3]. It works as a signaling particle in a range of processes, including cell metabolism, cell death and cell survival. Therefore, it plays a role in proliferation control [8]. Higher levels of NO can cause oxidative DNA damage [3] through alkylation, nitrosamine formation, DNA strand breaks or nitrosative deamination and inhibition of DNA repair systems [8]. The harmful effects of NO are typically observed in chronic inflammation, which upregulates NOS2 expression. There are some studies suggesting that inflammation plays a role in the initiation of cancer by the induction of neoplastic transformation and further progression of cancerogenesis [9]. The results of de Oliveira et al., 2017, support the inflammation significance in this process, suggesting that NSAIDs (non-steroidal anti-inflammatory drugs) can prevent or delay colorectal cancer [8]. An increased level of NO can cause tissue injuries [10], induce cytotoxicity and angiogenesis, which play a role in carcinogenesis [11]. Elevated iNOS (product of NOS2 gene) levels have been observed in a number of human cancers, including colorectal cancer [3,7,12], and it has often been a predictor of poor outcomes [12]. The amount of generated NO is influenced not only by the upregulation of NOS2 expression but also by certain genetic alterations of the NOS2 gene. Among several SNPs (single-nucleotide polymorphisms) described in the NOS2 gene, the rs2297518 is the most common in Europe [10]. It involves a G/A substitution in exon 16, which results in amino acid substitution Ser608Leu [3]. The amino acid substitution is located in the catalytic domain of the protein [10] and it may affect the catalytic activity of iNOS [6]. Therefore, the rs2297518 genotype is associated with a higher production of NO [6,10], although the SNP does not significantly change the NOS2 expression [6].

Colorectal cancer (CRC) is a heterogeneous disease, with its pathogenesis and prognosis largely dependent on stage, tumor grade, molecular profile, histopathological type and finally on the tumor location. The division of the intestine into the right (proximal) and left (distal) portions arises from distinct embryological origins. Cecum, ascending colon and most of the transverse colon originate from the midgut, whereas splenic flexure, sigmoid colon, descending colon and rectum originate from the hindgut. Colorectal cancer originates from the epithelial tissue of the colon and rectum. Depending on their location, these tumors differ in the aspect of their driving mutations, histology and disease progression [13,14,15]. The right-sided colorectal cancers (RCRCs) are frequently symptomless, often poorly differentiated or mucinous adenocarcinomas, but interestingly, the stage is lower compared to left-sided tumors (LCRCs). Molecular characteristics of most RCRCs include mutations in the DNA mismatch repair pathway, which results in microsatellite instability (MSI) and hypermutated cancers. These tumors are called MSI-high [15]. In the LCRCs, chromosomal instability (CIN) is commonly observed. It usually involves the APC, KRAS and p53 mutations and a high frequency of DNA copy number alterations. Tumors developing in the CIN pathway, also known as traditional pathway, have a polypoid morphology, easily detected with a colonoscopy in the early stages of carcinogenesis. This is one of the reasons for the higher 5-year survival rate for LCRC [15,16]. These tumors also differ in terms of metastatic patterns: LCRCs typically metastasize to the liver and lungs, while RCRCs show peritoneal and omental dissemination [17]. Overall, the 5-year survival rate (OS) for RCRCs is higher than that for LCRCs [12], and regarding rectal cancer it is even slightly higher than that for colon cancers. Cancers arising from the rectum have several different distinctive features, like risk factors or clinical manifestations. The rectum is exposed to a more concentrated fecal matter, a different pH and a gradient of hormone receptors [18]. Genetic variations affect the risk of colon and rectal carcinoma independently [19], which suggests a different genetic make-up of these malignancies.

In light of the above, CRCs should be considered more as a heterogeneous group of disease entities. Their pathogenesis is highly affected by the anatomical location of the tumor, which in turn affects their molecular, histological and clinical characteristics [15,20]. Hence, it is advisable to account for the location in genetic and epidemiological investigations to comprehensively grasp the diversity inherent to a complex condition like CRC.

The aim of this study was to determine the NOS2 polymorphism influence on colorectal cancer development with regard to the tumor localization. The research hypothesis proposes that the presence of allele A in the NOS2 rs2297518 genotype leads to an increase in NO levels, potentially promoting colorectal carcinogenesis. However, given the diverse properties of colorectal tumors, particularly their pathogenesis based on location, this project aims to test this hypothesis while stratifying patients into groups of right-sided, left-sided and rectal cancers.

## 2. Materials and Methods

### 2.1. Cohort Characteristic

This study was conducted in two clinical centers in Kielce, Poland: the Kielce Provincial Hospital and the Holy Cross Cancer Center. Patient inclusion criteria were age >18, giving informed consent for the study and colonoscopy examination. Exclusion criteria were genetic predisposition syndrome to CRC (FAP, HNPCC), CRC in family history, polyps in colon and pregnancy. Our study included 199 patients with colorectal cancer confirmed by pathological diagnosis of specimens collected during colonoscopy or surgery. All cases were reclassified according to the 8th edition UICC (Union for International Cancer Control), 2017 [21]. The control group consisted of 120 patients without colorectal cancer or polyps as confirmed by an endoscopic and/or histopathological examination. All patients signed written consent forms for genetic testing. Clinical data and blood samples were collected (test tubes with EDTA provided by Sarsted, Warszawska 25, 05-082 Stare Babice, Poland). Coded samples were frozen at –80 °C for genetic testing. The research protocol was approved on 3 June 2013 by the local Bioethics Commission (No. 5/2013) with the annotation on 4 November 2022. All procedures performed in the study followed the institution’s ethical standards, the Helsinki declaration and its later amendments.

### 2.2. Genotyping

Peripheral blood leukocytes were the material for genetic testing. The genomic DNA was extracted from blood samples using the automatic nucleic acid extractor and genomic DNA whole blood kit (Magcore^®^, RBC BioScience, New Taipei City, Taiwan). The purity and concentration of the isolated DNA were evaluated spectrophotometrically at 260 nm and 280 nm (Denovix, DS-11, Wilmington, DE, USA). Analysis of the NOS2 SNP rs2297518 was performed using the TaqMan^®^ genotyping Assays (Applied Biosystems, Foster City, CA, USA), according to manufacturer’s instructions, qPCR method—endpoint genotyping. PCR amplification using ≈10 ng of genomic DNA was performed with an initial step of 95 °C for 10 min followed by 50 cycles of 95 °C for 15 s (denaturation step) and 60 °C for 60 s (annealing and elongation step).

### 2.3. Statistical Analysis

The sample size (assuming dominant model) in the CRC and control groups was as-sensed as 223 and 114 (2:1 ratio), respectively, at a power of 80% and a two-sided alpha value of 5% under the hypothesis of an expected OR = 2 and minor allele frequency = 20% following Serdar’s recommendations [22]. Continuous data were described by means, standard deviations, medians, quartiles and range (minimum and maximum). Categorical data were summarized by frequencies and percentages. Group comparisons were performed using the chi-square or Fisher exact test for categorical variables, and Mann–Whitney test for continuous, non-normally distributed variables (normality of distributions was rejected by Shapiro–Wilk test). Odds ratios (OR) with 95% confidence intervals were calculated with logistic regression model. Hardy–Weinberg equilibrium was tested by chi-square goodness-of-fit test. A two-tailed *p*-value < 0.05 was considered statistically significant. All statistical analyses were performed using the R software package version 4.0.3.

## 3. Results

The demographic characteristic of the study population and the NOS2 genotyping results are shown in Table 1. The control and study groups do not differ statistically in terms of age and BMI. Analysis of all five genetic models did not reveal the influence of the A allele on CRC development [Table 2]. What is more, no statistically significant differences were observed in the frequency of the studied polymorphisms after adjustment for sex, age and BMI (Supplementary Materials). There is a male predominance in the CRC group (Table 1), but differences in tumor localization with respect to gender were not statistically significant [Table 3]. The NOS2 genotype distribution of the participants did not deviate from the Hardy–Weinberg equilibrium (*p* = 0.44). The total frequency of the minor allele A in the study population was 0.1928. The comparison of the frequency of the investigated polymorphism between the colorectal cancer group and the control group did not reveal statistically significant differences. However, a 4-percentage-point predominance of the wild-type genotype in the control group does indicate a tendency toward the more frequent occurrence of the investigated polymorphism in CRC patients [Table 1].

The stratification of colorectal cancer patients into subgroups based on tumor location revealed differences in tumor characteristics as well as in the frequency of the minor allele A in the NOS2 genotype [Table 2]. The distribution of histological tumor types significantly differed between the right-sided and left-sided locations (*p* = 0.0043), as well as between the right-sided and rectal locations (*p* = 0.0001). Mucinous adenocarcinoma was significantly more prevalent in right-sided cancers than in left-sided cancers (30.6% vs. 10.9%, *p* = 0.009) and more frequent than in rectal cancers (30.6% vs. 7.1%, *p* = 0.0003). Conversely, NOS (not otherwise specified) adenocarcinoma was more common in left-sided cancers (87.5% vs. 65.3%, *p* = 0.0049) and rectal cancers (92.9% vs. 65.3%, *p* < 0.0001) than in right-sided tumors. However, no association of the rs2297518 polymorphism with mucinous tumor type was observed in any of the NOS2 genetic models (Supplementary Materials).

The cancer grade and stage in terms of localization as determinants of colorectal cancer prognosis were examined in this study (Table 2).

The histological grade of CRC is assessed based on the degree of tumor differentiation (the percentage of glandular differentiation) in the tumor according to the World Health Organization (WHO) criteria. In the four-step scale, the lowest number—G1—corresponds to the “low-grade” tumor, which means that cancer cells are differentiated, rather similar to the healthy cells. The highest number—G4—corresponds to the “high-grade” tumor, which means that cancer cells are completely undifferentiated. In general, the lower the tumor’s grade, the better the prognosis [23].

Among diagnosed RCRCs, there are significantly more G3 grade tumors (16.3%) than in LCRCs (16.3% vs. 3.1%, *p* = 0.0185). The tumor–node–metastasis (TNM) staging system of the American Joint Committee on Cancer (AJCC) is the most widely used prognostic standard for CRC 2 and has been revised several times in recent decades to improve its prognostic performance and treatment suggestions for patients with CRC. The TNM system relies on the assessment of three components: the extent of the primary tumor (T), the absence or presence of regional lymph node metastases (N) and the absence or presence of distant metastatic lesions (M). This classification may be either clinical, based on evidence acquired before definitive treatment or pathological (pTNM), when intraoperative and surgical pathological data are available, which is a final result of tumor staging [24].

Higher-stage tumors were most frequently detected on the right side [Table 2]. However, due to the undefined metastasis in a considerable number of cases (pTNM classification), the observed differences do not allow for a reliable interpretation. According to Table 2, the rs2297518 polymorphism is significantly more frequent in right-sided cancers than in left-sided cancers (44.9% vs. 23.1%, *p* = 0.0137). The presence of allele A in the NOS2 genotype nearly triples the chances of developing cancer on the right side compared to the left side [OR = 2.72, 95%CI: 1.21–6.08, *p* = 0.0151].

This study also revealed a significant difference (23.1% vs. 40.0%, *p* = 0.0285) in the frequency of the investigated polymorphism between left-sided and rectal cancers. Among all three CRC locations, the wild-type genotype was most frequently observed in left-sided cancers (Table 2). The presence of allele A in the NOS2 genotype increases the chances of rectal cancer by over two times compared to left-sided cancers [OR = 2.22, 95%CI: 1.08–4.55, *p* = 0.0302].

## 4. Discussion

The characteristics of the study group reflect global epidemiological data regarding the incidence of CRC. According to global data, the incidence of this disease increases significantly after the age of 50 [25]. The median age of diagnosis of cancer in the study group is 65 years, and the control group was matched to this age. More than 90% of people diagnosed with CRC are 50 years old and over [15].

Based on the success of CRC screening in reducing CRC incidence and mortality, the US Preventive Services Task Force recommends screening of asymptomatic adults aged 50 through 75 [25]. However, CRC incidence and mortality rates in persons under age 50, termed early age onset CRC (EAO-CRC), have been increasing annually in the last ten years [26].

According to the National Cancer Database, the frequency of RCRC increases with age [27]. A number of studies confirm that RCRC occurs predominantly in females and older individuals, while LCRC is more common in males and tends to occur at an earlier age [15,27]. This is the rationale for conducting an analysis of the age of CRC onset depending on its location. In our study, consistent with the literature, a higher percentage of left-sided cancers was observed in younger individuals (<50 years old). However, due to the very small number of patients in this age group within the studied population, this difference was not statistically significant.

In the CRC group, there is a male predominance, which aligns with the disease’s population frequency in Europe and worldwide [28,29,30]. Additionally, gender also plays a role in the localization of the tumor. According to White et al., in their study on the UK population, the proportion of LCRC and rectal cancer cases is higher in males (54.6%) than females (43.5%). The proportion of RCRC cases is higher in females (27.0%) than males (19.5%), for whom they are harder to detect and diagnose [31]. The right-sided and left-sided parts of the colon are derived from two different embryonic tissues, with their own vascular supplies and biological environments, leading to differential sensitivity to sex hormones and consequent variation in carcinogenesis pathways between men and women [28].

The female sex is also more associated with hypermethylation, microsatellite instability, BRAF V600E mutation and CpG island methylator phenotype (CIMP)-high [31], which is consisted with the genetic architecture of RCRC [29] and leads to more aggressive forms of cancer [31]. However, in our study, no statistical differences in the frequency of rs2297518 between female and male groups of the CRC population were observed.

The total frequency of the minor allele A in the studied population is 0.1928, which is consistent with the NCBI (National Center for Biotechnology Information) database for the worldwide and European population, respectively, A = 0.1981 [32]. Genotype rs2297518, resulting in overproduction of NO, can generate oxidative stress in cells and through this mechanism it can promote cancerogenesis. This hypothesis was analyzed in a number of studies [11,33,34]. They showed an association of rs2297518 with prostate cancer in Caucasians and African Americans [11], non-Hodgkin’s lymphoma in Polish population [33] and gastric cancer in the Chinese population [34]. Slattery et al. (2012), in a study conducted on a population with similar demographic characteristics but a larger sample size, observed an association between rs2297518 and colon cancer [2]. The difference in the frequency of the investigated polymorphism observed in our study between the CRC group and the control group was not significant (*p* = 0.4643 in the dominant model). Nevertheless, when divided into groups based on the cancer location, statistical differences became evident (Table 2). Considering that there is a clear distinction between RCRC, LCRC and rectal cancer in terms of epidemiology, genetic pathways, clinical manifestations and also survival rates [12,17,35], researchers are placing more emphasis on the specific tumor location rather than on CRC as a whole. Since right-sided tumors likely develop through a different carcinogenic pathway than left-sided ones, and different genetic variants may affect the risk of RCRC and LCRC [20], which is reflected in our findings. In the studied population, the wild-type genotype is statistically more prevalent in left-sided cancers compared to right-sided ones (*p* = 0.0137) and compared to rectal cancers (*p* = 0.0285). This is consistent with a large clinical GWAS (Genome Wide Association Study) conducted by Huyghe and colleagues (2021), which revealed that genetic risk variants for CRC are partly distinct for proximal and distal CRC [36]. Studies on the genetic architecture of tumors themselves also identified a significantly higher mutation burden and median oncogenic mutation burden in tumors originating from the right side of the colon, in contrast to the left side [14,17].

In the studied population, the genetic research outcomes correspond with the clinical diversity of these tumor types. The mucinous adenocarcinoma type was statistically more frequently observed on the right side: 30.6% in RCRCs compared to 10.9% in LCRCs (*p* = 0.0048) (Table 2). This is consistent with global reports indicating that tumors found in RCRC often manifest as mucinous adenocarcinomas [15,37,38,39]. Shimada and colleagues (2017) obtained similar results regarding the diversity of tumor types and grading with respect to the location of the CRC. In both our study and theirs, among diagnosed RCRC cases, there were significantly more mucinous-type and G3-grade tumors [14].

Moreover, the right-sided colorectal cancers are often MSI-high tumors, which carry more immunogenic mutations and harbor an increased number of neoantigens [15]. Recent genomic studies suggested that a high tumor mutation burden confers an increased immune reaction to tumors [13,40]. NOS2 is induced in these immunological stimulated tissues and produces large amounts of NO [4,6], which is even higher in the case of the studied polymorphism rs2297518 [3]. Substantially elevated levels of NO may, in turn, foster carcinogenesis, particularly for this CRC location. This could partially account for the higher frequency of the NOS2 polymorphism among individuals with tumors in the proximal part of the colon compared to the distal part.

Numerous recent studies suggest that oxidative stress may play a significant role in the pathogenesis of CRC in terms of localization. Janion et al. observed the higher mean TOS (total oxidative status) level in patients with a right-sided tumor location compared to patients with a left-sided tumor location. It was also found that the right-sided location is linked with enhanced lipid peroxidation processes compared with the left-sided location and the rectum [29]. Analyzed in our study, the NOS2 gene’s polymorphism can increase the oxidative stress in the epithelium of the colon through the enhanced production of NO [10]. The obtained results indicate that this polymorphism occurs more frequently in right-sided cancers, thus supporting the hypothesis that oxidative stress favors the development of cancer in this location.

## 5. Conclusions

Our study results confirm the distinction between right-sided CRC and left-sided CRC in terms of grade, stage, histopathological type and also molecular disturbances. Considering RCRC, LCRC and RC as separate entities is crucial for developing effective prophylactic programs, therapy regimes and better treatment options for patients. We have demonstrated that the NOS2 polymorphism rs2297518 significantly increases the risk of RCRC compared to LCRC. This is one of the elements of genetic predisposition for the development of right-sided or left-sided tumors with specific clinical properties and different prognoses. Understanding the genetic risk factors is crucial for the development of distinct screening and prophylactic programs tailored to individuals with a specific genetic profile. Such personalization of prevention and treatment could potentially reduce the incidence of colon and rectal cancers and simultaneously increase patient survival rates.

## 6. Study Limitations

We acknowledge that the lack of statistical significance in demonstrating differences in the observed frequency of the rs2297518 polymorphism between the study and control groups could be attributed to the relatively small population size, although the group sizes were comparable to other published reports. The dietary pattern could also be confounding a factor; however, obtaining reliable data on this issue through questionnaire-based surveys is very unreliable, especially in the case of patients with colorectal cancer, wherein the diagnosis typically enforces dietary changes. Nevertheless, it would be valuable to consider the dietary factor and its potential interactions with the genetic polymorphism in a larger group of the Caucasian population in the future to confirm the obtained results.

## Figures and Tables

**Table 1:** Demographic characteristic and NOS2 genotyping results of the study population.

	Control (N = 120)	CRC (N = 199)	*p*-Value
Sex			0.0177
Female	64 (53.3%)	79 (39.7%)	
Male	56 (46.7%)	120 (60.3%)	
Age			0.0864
Mean (SD)	62.14 (11.67)	64.54 (8.40)	
Median (Q1, Q3)	64.00 (54.00, 70.25)	65.00 (59.00, 71.00)	
Range	38.00–94.00	38.00–81.00	
BMI			0.8069
N-Miss *	2	1	
Mean (SD)	27.33 (4.56)	27.42 (4.39)	
Median (Q1, Q3)	26.85 (23.72, 30.35)	26.90 (24.45, 29.70)	
Range	18.40–38.40	17.50–42.20	

* N-miss—number of missing data.

**Table 2:** NOS2 polymorphism frequency in CRC and control groups.

	Controls			CRC					
	n	%	n	%	OR	Lower	Upper	*p*-Value	AIC
Codominant								0.6616	427.6
G/G	82	68.3	128	64.3	1.00				
A/G	34	28.3	61	30.7	1.15	0.70	1.90		
A/A	4	3.3	10	5.0	1.60	0.49	5.28		
Dominant								0.4631	425.9
G/G	82	68.3	128	64.3	1.00				
A/G-A/A	38	31.7	71	35.7	1.20	0.74	1.94		
Recessive								0.4665	425.9
G/G-A/G	116	96.7	189	95.0	1.00				
A/A	4	3.3	10	5.0	1.53	0.47	5.01		
Over dominant								0.6601	426.3
G/G-A/A	86	71.7	138	69.3	1.00				
A/G	34	28.3	61	30.7	1.12	0.68	1.84		
log-Additive								0.3838	425.7
0, 1, 2	120	37.6	199	62.4	1.20	0.80	1.79		

**Table 3:** Characteristics of CRC group and tumors depending on their location.

	Rectal Cancer (N = 85)	Left-Sided CRC (N = 65)	Right-Sided CRC (N = 49)	Rectal vs. Left-Sided *p*-Value	Rectal vs. Right-Sided *p*-Value	Left vs. Right-Sided *p*-Value
Sex				0.5012	0.8969	0.6442
female	32 (37.6%)	28 (43.1%)	19 (38.8%)			
male	53 (62.4.2%)	37 (56.9%)	30 (61.2%)			
age				1	0.4148	0.389
<50	5 (5.9%)	4 (6.2%)	1 (2.0%)			
≥50	80 (94.1%)	61 (93.8%)	48 (98.0%)			
Histological type				0.3788	0.0001	0.0043
N-Miss *	1	1	0			
mucinous	6 (7.1%)	7 (10.9%)	15 (30.6%)			
mucinous and solid	0 (0.0%)	1 (1.6%)	0 (0.0%)			
solid	0 (0.0%)	0 (0.0%)	2 (4.1%)			
NOS **	78 (92.9%)	56 (87.5%)	32 (65.3%)			
Grade				0.1781	0.3627	0.0368
G1	2 (2.4%)	6 (9.2%)	1 (2.0%)			
G2	70 (82.4%)	54 (83.1%)	37 (75.5%)			
G3	6 (7.1%)	2 (3.1%)	8 (16.3%)			
Gx	7 (8.2%)	3 (4.6%)	3 (6.1%)			
pT				0.2054	0.4876	0.4206
N-Miss *	18	19	10			
0	1 (1.5%)	0 (0.0%)	0 (0.0%)			
1	5 (7.5%)	5 (10.9%)	1 (2.5%)			
2	13 (19.4%)	3 (6.5%)	4 (10.0%)			
3	36 (53.7%)	25 (54.3%)	25 (64.1%)			
4	12 (17.9%)	13 (28.3%)	9 (23.1%)			
pN				0.2783	0.9415	0.321
N-Miss *	19	21	9			
N0	31 (47.0%)	23 (52.3%)	19 (47.5%)			
N1a	18 (27.3%)	16 (36.4%)	10 (25.0%)			
N1b	5 (7.6%)	2 (4.5%)	5 (12.5%)			
N2a	6 (9.1%)	0 (0.0%)	2 (5.0%)			
N2b	4 (6.1%)	1 (2.3%)	3 (7.5%)			
Nx	2 (3.0%)	2 (4.5%)	1 (2.5%)			
pM				0.1519	0.0084	0.3385
N-Miss *	31	30	24			
0	0 (0.0%)	0 (0.0%)	2 (8.0%)			
1	0 (0.0%)	2 (5.7%)	2 (8.0%)			
x	54 (100.0%)	33 (94.3%)	21 (84.0%)			
Dominant model_NOS2_rs2297518				0.0285	0.5798	0.0137
Homozygous GG (WT ***)	51 (60.0%)	50 (76.9%)	27 (55.1%)			
Homozygous AA or Heterozygous AG	34 (40.0%)	15 (23.1%)	22 (44.9%)			

N-miss *—number of missing data, NOS **—not otherwise specified; WT ***—wild type.

## Data Availability

The data presented in this study are available upon request from the corresponding author.

## Supplementary Materials

The following supporting information can be downloaded at: https://www.mdpi.com/article/10.3390/jcm13040937/s1, Table S1. NOS2 gene polymorphism frequency in mucinous and non-mucinous CRC. Table S2. Multivariant analysis of NOS2 polymorphism on CRC risk adjusted by gender, age (<50, 50+) and BMI (normal, overweight, obesity).
