# Supplementary material for: NOS2 Polymorphism in Aspect of Left and Right-Sided Colorectal Cancer
Source: J Clin Med. 2024 Feb 6;13(4):937. doi: 10.3390/jcm13040937 (PMC10888565; doi:10.3390/jcm13040937)
Supplement: Supplementary file 1 [file jcm-13-00937-s001.zip › jcm-2820733-supplementary.pdf]

Journal of Clinical Medicine

MDPI

Supplementary materials:

Table S1. NOS2 gene polymorphism frequency in mucinous and non-mucinous CRC

	**Non-mucinous**		**Mucinous**						
	n	%	n	%	OR	lower	upper	p-value	AIC
Codominant								0.9021	170.4
G/G	108	64.3	19	65.5	1.00				
A/G	51	30.4	9	31.0	1.00	0.42	2.37		
A/A	9	5.4	1	3.4	0.63	0.08	5.28		
Dominant								0.8980	168.6
G/G	108	64.3	19	65.5	1.00				
A/G-A/A	60	35.7	10	34.5	0.95	0.41	2.17		
Recessive								0.6500	168.4
G/G-A/G	159	94.6	28	96.6	1.00				
A/A	9	5.4	1	3.4	0.63	0.08	5.18		
Overdominant								0.9417	168.6
G/G-A/A	117	69.6	20	69.0	1.00				
A/G	51	30.4	9	31.0	1.03	0.44	2.42		
log-Additive								0.7881	168.6
0,1,2	168	85.3	29	14.7	0.91	0.46	1.81		

Table S2. Multivariant analysis of NOS2 polymorphism on CRC risk adjusted by gender, age (<50, 50+) and BMI (normal, overweight, obesity)

	**controls**		**CRC**						
	n	%	n	%	**adjusted OR**	lower	upper	p-value	AIC
**Codominant**								0.8959	410.2
G/G	80	67.8	127	64.1	1.00				
A/G	34	28.8	61	30.8	1.13	0.67	1.91		
A/A	4	3.4	10	5.1	1.11	0.33	3.78		
**Dominant**								0.6396	408.2
G/G	80	67.8	127	64.1	1.00				
A/G-A/A	38	32.2	71	35.9	1.13	0.68	1.87		
**Recessive**								0.9145	408.4
G/G-A/G	114	96.6	188	94.9	1.00				
A/A	4	3.4	10	5.1	1.07	0.32	3.58		
**Overdominant**								0.6634	408.2
G/G-A/A	84	71.2	137	69.2	1.00				
A/G	34	28.8	61	30.8	1.12	0.67	1.89		
**log-Additive**								0.6648	408.2
0,1,2	118	37.3	198	62.7	1.10	0.72	1.69		
